# Molybdenum carbide/Ni nanoparticles-incorporated carbon nanofibers as effective non-precious catalyst for urea electrooxidation reaction

**DOI:** 10.1038/s41598-022-26975-5

**Published:** 2022-12-30

**Authors:** Nasser A. M. Barakat, Marwa A. Ali

**Affiliations:** Chemical Engineering Department, Faculty of Engineering, Minia University, El-Minia, 61519 Egypt

**Keywords:** Energy science and technology, Materials science

## Abstract

In this study, molybdenum carbide and carbon were investigated as co-catalysts to enhance the nickel electro-activity toward urea oxidation. The proposed electrocatalyst has been formulated in the form of nanofibrous morphology to exploit the advantage of the large axial ratio. Typically, calcination of electropsun polymeric nanofibers composed of poly(vinyl alcohol), molybdenum chloride and nickel acetate under vacuum resulted in producing good morphology molybdenum carbide/Ni NPs-incorporated carbon nanofibers. Investigation on the composition and morphology of the proposed catalyst was achieved by XRD, SEM, XPS, elemental mapping and TEM analyses which concluded formation of molybdenum carbide and nickel nanoparticles embedded in a carbon nanofiber matrix. As an electrocatalyst for urea oxidation, the electrochemical measurements indicated that the proposed composite has a distinct activity when the molybdenum content is optimized. Typically, the nanofibers prepared from electrospun nanofibers containing 25 wt% molybdenum precursor with respect to nickel acetate revealed the best performance. Numerically, using 0.33 M urea in 1.0 M KOH, the obtained current densities were 15.5, 44.9, 52.6, 30.6, 87.9 and 17.6 mA/cm^2^ for nanofibers prepared at 850 °C from electropsun mats containing 0, 5, 10, 15, 25 and 35 molybdenum chloride, respectively. Study the synthesis temperature of the proposed composite indicated that 1000 °C is the optimum calcination temperature. Kinetic studies indicated that electrooxidation reaction of urea does not follow Arrhenius’s law.

## Introduction

Scientists have discovered that urea pollution can cause ocean algae to develop a fatal poison known as domoic acid^1^. Paradoxically, urea can be manipulated as a non-toxic, non-flammable hydrogen-carrying molecule with an energy density of 16.9 MJ L^−1^ (approximately 10 times more than hydrogen). Moreover, compared to water, electrolysis of urea consumes lower electrical power^2^. Theoretically, hydrogen extraction from urea is a straight forward process as it depends on an exothermic reaction according to the following equations^2–6^:

1 $$ \begin{array}{*{20}c} {{\text{Anode}}:\;{\text{CO}}\left( {{\text{NH}}_{2} } \right)_{2} + 6{\text{OH}}^{ - } \to {\text{N}}_{2} + 5{\text{H}}_{2} {\text{O}} + {\text{CO}}_{2} + 6{\text{e}}} & {{\text{E}}^{0} = - \;0.746\;{\text{V}},} \\ \end{array} $$

2 $$ \begin{array}{*{20}c} {{\text{Cathode}}:\;6{\text{H}}_{2} {\text{O}} + 6{\text{e}} \to 3{\text{H}}_{2} + 6{\text{OH}}^{ - } } & {{\text{E}}^{0} = - \;0.829\;{\text{V}},} \\ \end{array} $$

3 $$ \begin{array}{*{20}c} {{\text{Overall}}:\;{\text{CO}}\left( {{\text{NH}}_{2} } \right)_{2} + {\text{H}}_{2} {\text{O}} \to {\text{N}}_{2} + 3{\text{H}}_{2} + {\text{CO}}_{2} } & {{\text{E}}^{0} = - \;0.083\;{\text{V}}.} \\ \end{array} $$

However, due to the high overpotentials over the reported electrodes, there is no any known anode material could achieve the task without power addition. Beside the low required energy (ca. 0.37 V) compared to water (1.23 V), there are other advantages for hydrogen extraction from urea electrolysis: (1) producing non-self-ignited gas mixture due to absence of oxygen, (2) converting the nitrogen pollution in the wastewater to an environmentally safe product; N_2_ and (3) arousing the researchers to develop new electrode materials having low overpotentials^7^.

Nickel attracts the attention of the researchers as an anode material in the urea electrolysis cell from the biological degradation of urea by urease. This enzyme consists of two Ni^+2^ attached two water molecules and bridging hydroxide group^8,9^. A vast number of studies have demonstrated that, in an alkaline media, nickel and nickel-based compounds are oxidised to nickel’s active state (NiOOH) and subsequently operates as a urea oxidation reaction (UOR) catalyst^10,11^. However, the electrocatalytic activity of the unmodified nickel does not meet the minimum requirements to be an applicable anode which could be translated as poor formation of the required active sites. Enhancing the electrocatalytic activity of nickel toward urea electrooxidation has been conducted in two main strategies; shape development and invoking co-catalyst(s). In the first route, the transition metal has been formulated either in its pristine or metal hydroxide (Ni(OH)_2_). In this regard, several nanostructural formulations have been investigated including nanowire arrays^12^, nanomeshes^13^, nanoribbons^14^, nanoflakes^15^ and nanosheets^16^.

As a co-catalyst, several metallic and non-metallic species have been investigated including cobalt^17,18^, iron^19^, manganese^20,21^, tin^2^, tungsten^22^, nitrogen^23^, phosphorous^24,25^, graphene^5,26^, CNTs^27,28^, and carbon nanofibers^29^. The main function of the co-catalyst is either decreasing the onset-potential and/or enhancing the generated current density of the urea electro-oxidation reaction. For these two objectives, molybdenum draws the attention of the researchers as a promised element for enhancing the activity of the nickel-based materials^30^. For instance, Yang et al.^31^ introduced nickel-molybdenum oxide (NiMoO_4_) nanorods for urea oxidation using simple hydrothermal and low-temperature heat treatment. When the precursors’ Ni/Mo ratio is 2, the resulting catalyst has fast kinetics, low electron transfer resistance, and a low Tafel slope for urea oxidation. Yu et al.^32^ describe a porous rod-like NiMoO_4_ with high metal element oxidation states that enables very effective UOR electrocatalysis and can be easily made by annealing solid NiMoO_4_*x*H_2_O as a starting precursor in Ar. When the shielding gas is changed from Ar to H_2_/Ar, the resulting Ni/NiO/MoO*x* nanocomposite exhibits platinum-like activity for the hydrogen evolution process (HER) in alkaline electrolytes. Shi et al.^33^ used a simple reduction technique to synthesize Ni–Mo/graphene and then studied the efficacy of urea electrooxidation. Ni_2_Mo/Gr demonstrated exceptional performance, including high current density and long-term stability, due to structural activity and electron effect. Beside the oxide and zero-valent forms, MoS_2_/Ni_3_S_2_ catalyst has been reported as a new 3D-heteropore dual-function catalyst which exhibits low urea electrolysis cell voltage^34^. Overall, molybdenum doping has shown effective delay polarisation and increase the range of electric oxidation potential. However, the content as well as the molybdenum chemical state have to be optimized as it strongly affects the site distribution of the surface Ni^3+^ electroactive centre^35,36^. The heterogeneous catalytic electrooxidation reactions using a solid catalyst are considered a combination between adsorption and chemical reaction processes. Consequently, immobilization of the functional electrocatalysts on carbon supports distinctly improves the performance. In this regard, carbon nanostructures such as graphene^26^, carbon nanotubes^28^ and carbon nanofibers^37^ were widely used as supports due to the extreme surface area which promotes the adsorption process.

In this study, influence of utilizing molybdenum carbide (Mo_2_C) as a co-catalyst in Ni NPs-incorporated carbon nanofibers has been investigated by synthesizing molybdenum carbide/nickel NPs-incorporated carbon nanofibers (Mo_2_C/Ni-incorporated CNFs) with different co-catalyst contents. Compared to other nanostructures, the large axial ratio of the nanofibrous morphology distinctly enhances the electron transfer rate which positively impacts the electrocatalytic activity^38^. The proposed catalyst has been synthesized by calcination of electrospun nanofibers composed of nickel acetate, molybdenum chloride and poly(vinyl alcohol) under vacuum atmosphere. The utilized physiochemical characterizations confirm decomposition of the metal precursors to zero-valent nickel and molybdenum carbide, and graphitization of the used polymer to produce carbon nanofibers embedding the crystalline metallic nanoparticles. Electrochemical measurements emphasize the high activity of the new composite.

## Experimental

### Materials

The used chemicals have been utilized as-received without any further treatment. The used polymer to prepare the electrospun solution (PVA, poly(vinyl alcohol), MW = 65.000 g/mol) was purchased from DC Chemical Co., South Korea. Nickel acetate tetrahydrates (Ni(Ac)_2_, Ni(CH_3_COO)_2_·4H_2_O, 99.99 purity) and molybdenum chloride (MoCl_2_, purity 99.99%) were obtained from Sigma Aldrich, USA. Deionized water was utilized as solvent.

### Preparation of the nanofibers

Ni(Ac)_2_/PVA aqueous stock was prepared by mixing 10 wt% PVA and 20 wt% Ni(Ac)_2_ aqueous solutions in a 3:1 weight ratio. The mixture was stirred for 5 h at 50 °C to get full poly(condensation) of the acetate ion. Later on, specific amounts of molybdenum chloride were dissolved in the minimum amount of de-ionized water and mixed with certain amounts of the prepared Ni(Ac)_2_/PVA solution to prepare different solutions having 0, 5, 10, 15, 25 and 35 wt% of MoCl_2_ compared to Ni(Ac)_2_. The electrospinning procedure was carried out at a voltage of 20 kV in a room setting with a distance of 15 cm between the syringe and the revolving drum collector. After vacuum drying of the electrospun mats, a 5-h holding time calcination process was performed under vacuum at various temperatures (700, 850, and 1000 °C).

### Characterization

Scanning electron microscopy was used to validate the nanofibrous morphology (SEM and FESEM, Hitachi S-7400, Japan). X-ray diffraction was used to investigate the chemical composition of the produced nanostructures (XRD, Rigaku, Japan). Potentiostats were used to conduct the electrochemical tests (VersaStat 4, USA). A three-electrode cell configuration was used, with a working electrode of glass carbon electrode (GCE), a reference electrode of Ag/AgCl, and a counter electrode (CE) of Pt wire. The working electrode was made by smearing 15 μl of catalyst ink onto the GCE’s active surface. The catalyst ink was made by dispersing 2 mg of the functional ingredient in a solution of 20 µl Nafion and 400 µl isopropanol. After deposition, the electrodes were dried at 80 °C for 30 min^39^. To learn more about the surface composition, X-ray Photoelectron Spectroscopy analysis (XPS, AXIS-NOVA, Kratos Analytical Ltd, UK) was used. The following circumstances were used for the XPS analysis: 6.5 × 10^–9^ Torr for the base pressure, 20 eV for the resolution (Pass Energy), and 0.05 eV/step for the scan step.

## Results and discussion

### Crystalline structure, surface morphology, and composition of the prepared materials

**Figure 1:**
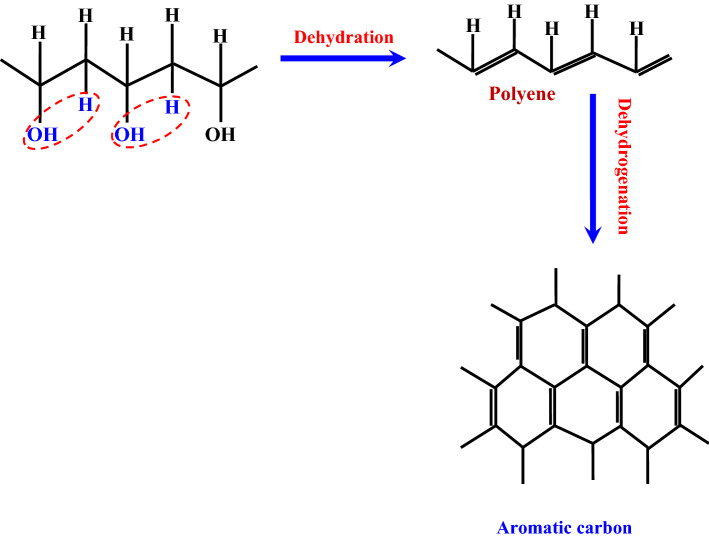
Schematic diagram for the expected optimum destructive of the PVA^41^.

PVA has significant high carbon content when compared to other vinyl polymers. However, because PVA melts and decomposes into volatile low molecular weight molecules at low temperatures, using it to fabricate carbon nanofibers is uncommon due to the difficulties of attaining acceptable shape and/or a high carbon yield^40^. To solve this problem, two primary tactics have been used: (1) pretreatment before the carbonization process, and (2) the use of certain catalysts during the heat treatment process to improve graphitization. Both procedures rely on cycling the PVA straight chain, which results in high melting point compounds that increase the graphitization process significantly. Figure 1 shows a conceptual representation of the best destructive disintegration of PVA to get the highest yield^41^. Dehydration and dehydrogenation methods may be used to extract aromatic carbon from the PVA straight chain, as demonstrated.

Utilizing nickel acetate as precursor has a dual function. First, under the inert atmosphere, this precursor fully decomposed to zero-valent nickel instead the expected nickel oxides. As it was proved by many researchers, heating this salt in an inert atmosphere leads to abnormal decomposition of the acetate anion to generate reducing gases (namely, carbon mono oxide and hydrogen) which results in producing of pure metal^42,43^. Formation of pure nickel was described by the following equations:

4 $$ {\text{Ni}}\left( {{\text{CH}}_{{3}} {\text{COO}}} \right)_{{2}} \cdot {\text{4H}}_{{2}} {\text{O}} \to 0.{\text{86Ni}}\left( {{\text{CH}}_{{3}} {\text{COO}}} \right)_{{2}} \cdot 0.{\text{14Ni}}\left( {{\text{OH}}} \right)_{{2}} + \, 0.{\text{28CH}}_{{3}} {\text{COOH }} + { 3}.{\text{72H}}_{{2}} {\text{O,}} $$

5 $$ 0.{\text{86 Ni}}\left( {{\text{CH3COO}}} \right)_{{2}} \cdot 0.{\text{14Ni}}\left( {{\text{OH}}} \right)_{{2}} \to {\text{NiCO}}_{{3}} + {\text{ NiO }} + {\text{ CH}}_{{3}} {\text{COCH}}_{{3}} + {\text{ H}}_{{2}} {\text{O}}, $$

6 $$ {\text{NiCO}}_{{3}} \to {\text{NiO }} + {\text{ CO}}_{{2}} , $$

7 $$ {\text{NiO }} + {\text{ CO}} \to {\text{Ni }} + {\text{ CO}}_{{2}} . $$

Second, nickel acetate has a polycondensation tendency which distinctly maintains the naofibrous morphology during the calcination process. The polycondensation reaction can be explained as follow^44^.8where M is the nickel atom. Therefore, the utilized precursors initially formed a good gel with the used polymer which facilitates the electrospinning process and consequently produces good morphology nanofibers. Moreover, formation of pristine nickel enhances the thermal stability of the utilized polymer during the calcination process^41,45^.

**Figure 2:**
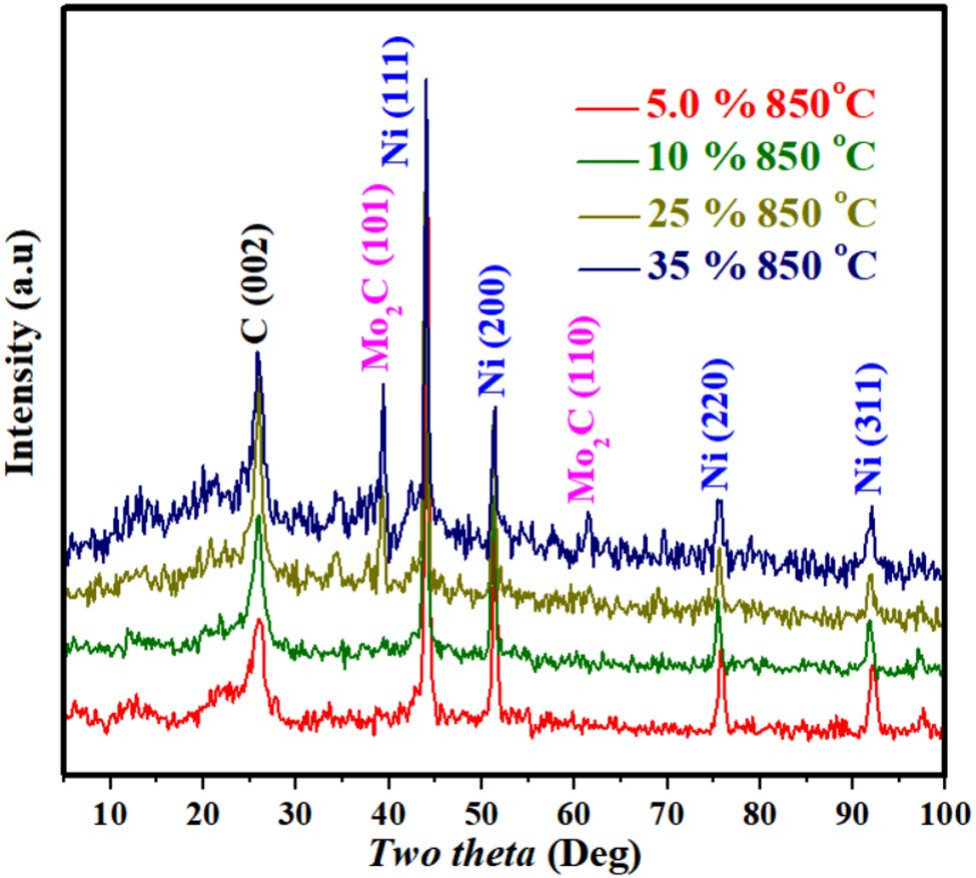
XRD results for the obtained powder after calcination at 850 °C of electrospun nanofiber mats prepared from sol–gel containing different amount of molybdenum precursor contents.

X-ray diffraction analysis is a powerful and trustable tool to check the chemical composition of the crystalline materials. Figure 2 displays the XRD patterns for selected samples after the calcination process. The results supported the aforementioned discussion about the decomposition track of nickel acetate. As shown, the representative peaks of the zero-valent nickel clearly appeared with all formulations. Typically, the observed strong peaks at 2θ values of 44.30°, 51.55°, 76.05° and 92.55° corresponding to (111), (200), (220) and (311) crystal planes, respectively confirm the formation of pure nickel (JCDPS# 04-0850). Moreover, the successful graphitization of the used polymer was also proved by the broad peak at 2θ of 26.3° corresponds to an experimental *d* spacing of 3.37 Å indicating presence of graphite-like carbon (graphite-2H, *d* (002), JCPDS#; 41-1487). Therefore, it can be claimed that the formed nickel catalyzes graphitization of PVA. On the other hand, molybdenum has combined with the formed carbon to form a thermally stable compound; molybdenum carbide (Mo_2_C). However, due to evolution of the reduced gases, the molybdenum has been formed in its lowest oxidation number (II)^46^. The Mo_2_C indexed peaks in the graph match the standard peaks of molybdenum carbide in International Centre for Diffraction Data Sample (JCPDS); # 35-0787. The representative peaks get strong with increasing the molybdenum precursor in the samples as shown in the figure.

**Figure 3:**
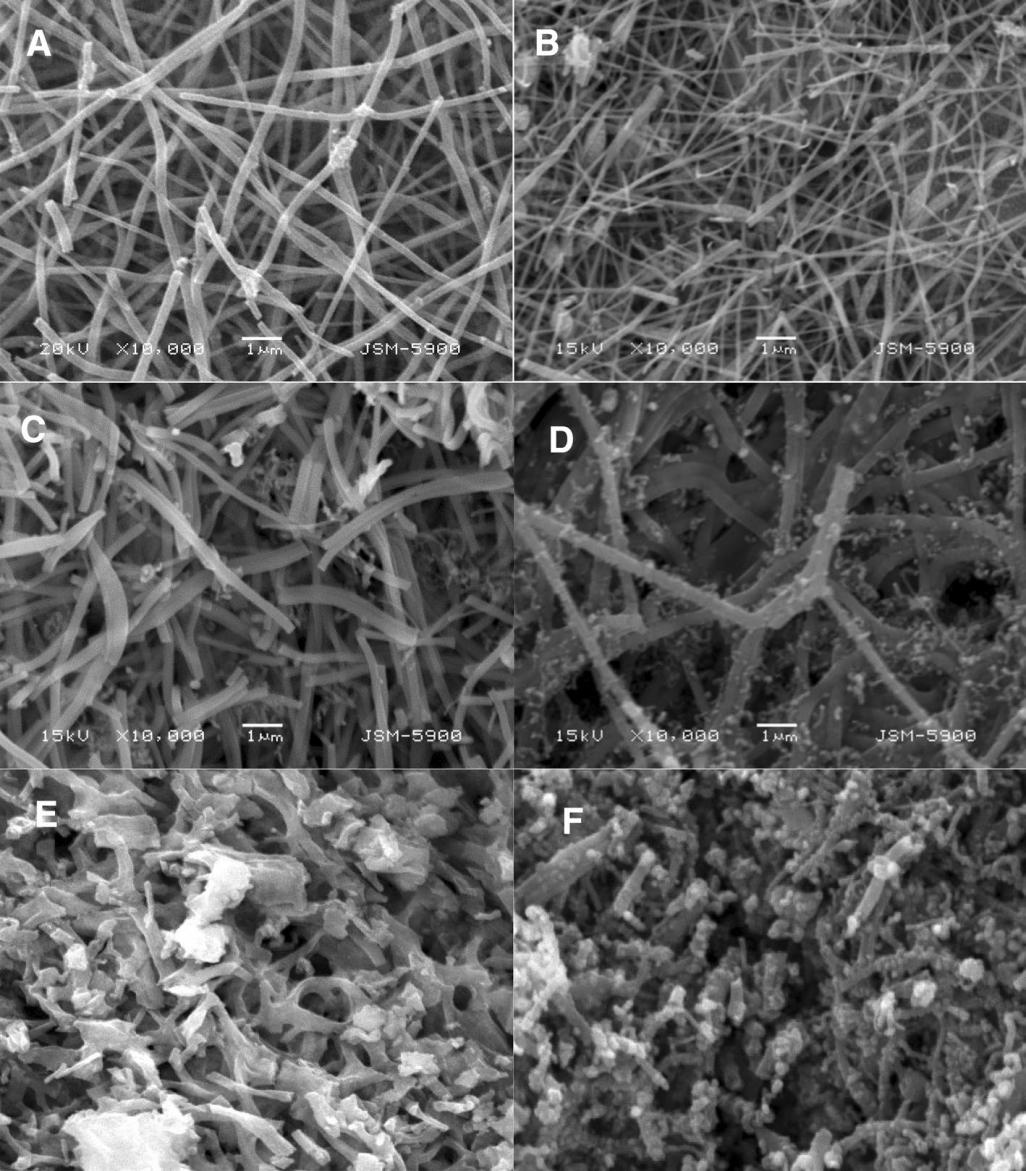
SEM images for the produced Mo_2_C–Ni–C composite nanofibers prepared at 850 °C calcination temperature and from original solution having 0 (**A**), 10 (**B**), 15 (**C**) and 35 (**D**) molybdenum chloride. Panels (**E,F**) display SEM images of the 35%-sample calcined at 700 and 1000 °C, respectively.

Figure 3 displays SEM images of randomly selected samples after performing the heat treatment process; 0 (Fig. 5A), 10 (Fig. 5A), 15 (Fig. 5A) and 35 (Fig. 5A) wt% MoCl_2_ samples prepared at 850 °C. From these panels, it can be concluded that, addition of molybdenum chloride, within the proposed range in this study, to the electrospun solution did not affect electrospinning possibility of the prepared solutions. Moreover, the initial morphology is thermally stable; subjecting the prepared electrospun nanofibers to high temperature treatment process did not annihilate the nanofibrous morphology. Figure 3E,F represent SEM image for the obtained power after calcination of 35%-sample at 700 and 1000 °C, respectively. Compared to the nanofibers prepared at 850 °C from the same electrospun solution (Fig. 3D), it can be stated that, using relatively low calcination temperature (700 °C) results in producing smooth surface nanofibers. However, increasing the calcination temperature to 850 °C led to form small nanoparticles attaching the main nanofibers. Further increase in the calcination temperature (to 1000 °C) results in increasing the number and size of the nanoparticles as well as decreasing the nanofibers axial ratio; Fig. 3F. It is worth mentioning that all formulations reveal almost similar results regardless the utilized Mo content. Maintaining the nanofibrous morphology can be attributed to the polycondensation feature of nickel acetate (Eq. 8) as well as the smart graphitization of the used polymer.

**Figure 4:**
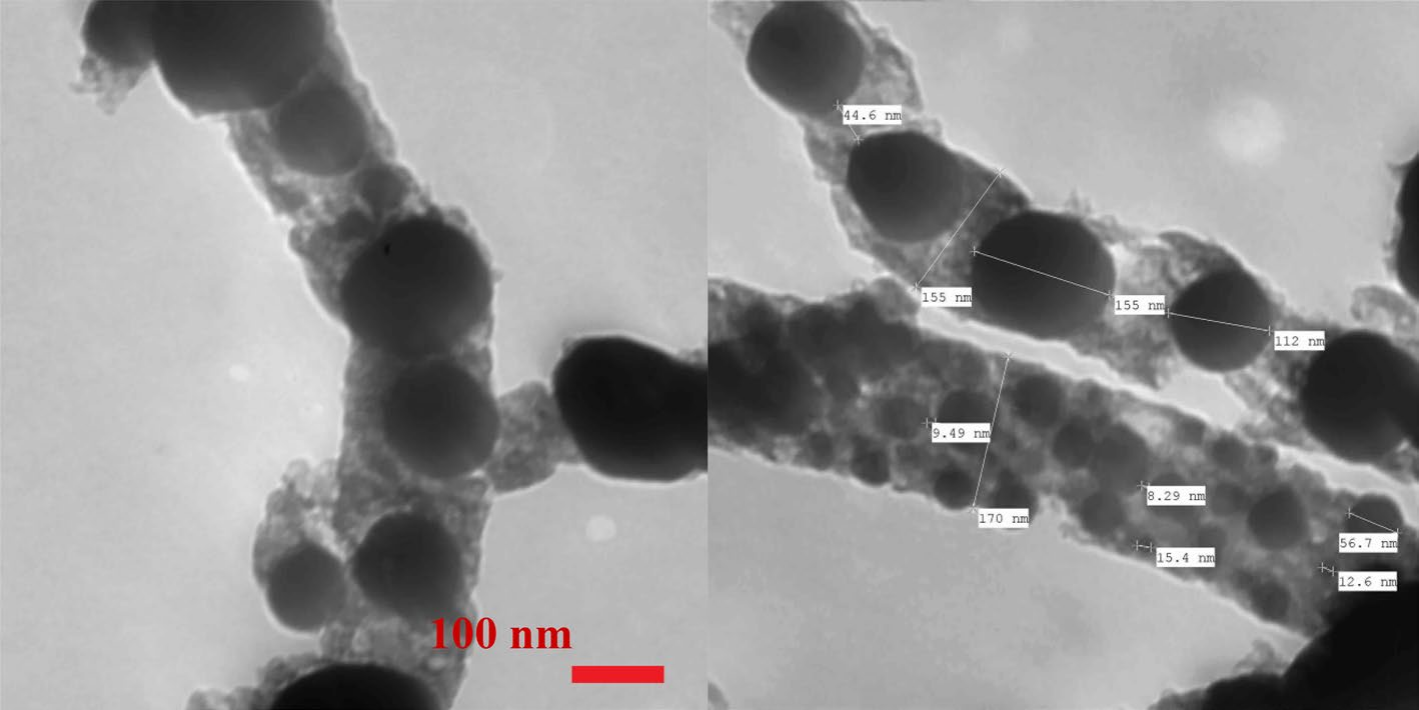
TEM image of Mo_2_C/Ni/graphite composite nanofibers prepared from an electrospun solution containing 10% molybdenum precursor at 850 °C.

Transmission electron microscopy (TEM) is an authorized analytical technique to detect the internal structure of the nanomaterials. Figure 4 demonstrates normal TEM image of the produced nanofibers after calcination of 10 wt% sample at 850 °C. The images can build a solid conclusion about the internal structure of the produced nanofibers. In TEM analysis, the dark areas represent crystalline materials due to the high refection of the used electron beams. Therefore, it can be alleged that the dark appeared dots represent the inorganic materials counterpart in the prepared nanofibers while the gray matrix is the detected graphite in the XRD analysis. Accordingly, the utilized physicochemical characterizations deduced that the proposed preparation methodology leads to prepare Mo_2_C/Ni NPs‒incorporated carbon nanofibers as a final product.

**Figure 5:**
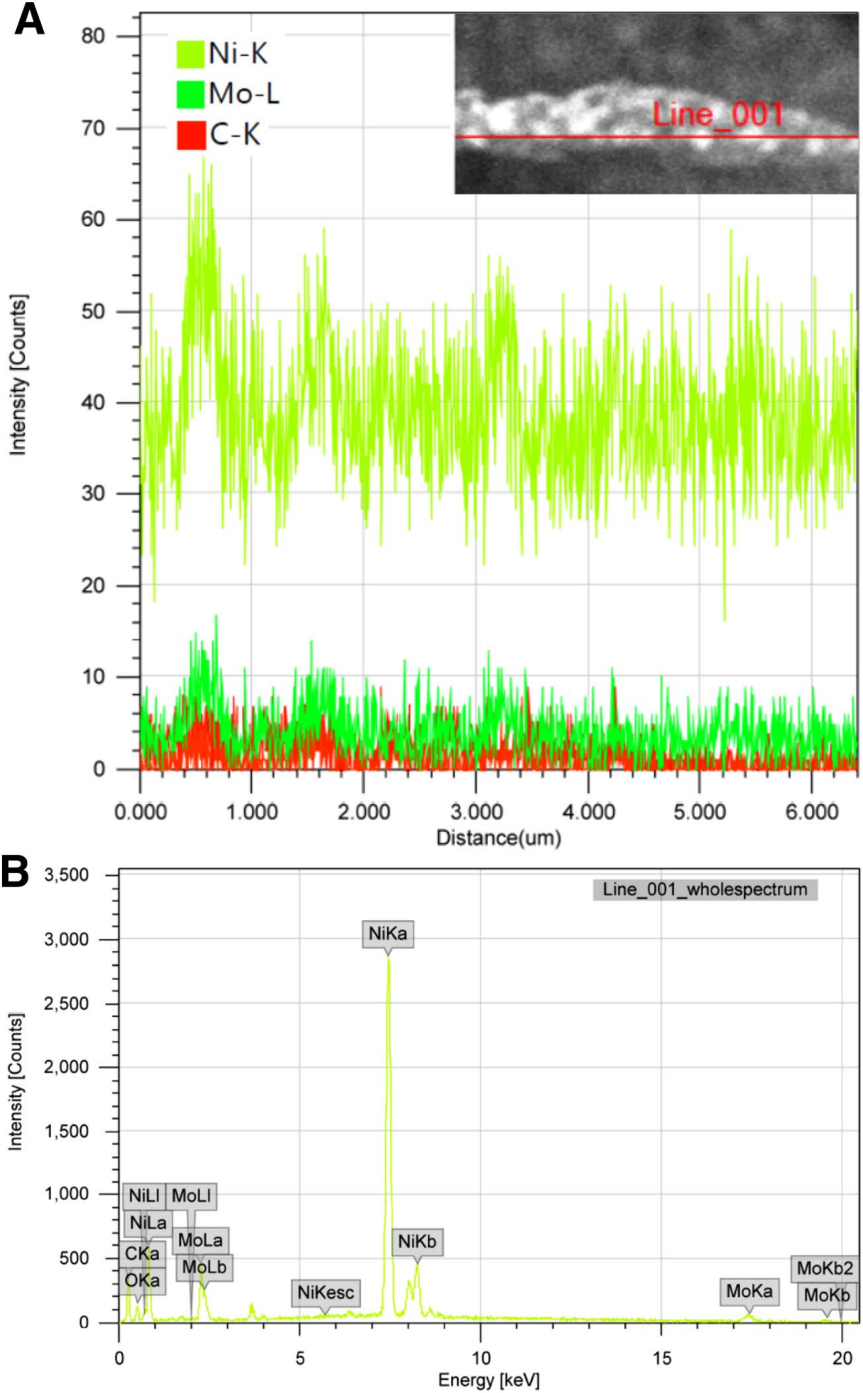
Line elemental mapping; (**A**) and EDX analysis; (**B**) for the produced Mo_2_C/Ni-incorporated carbon nanofibers from 25 wt% molybdenum chloride electrospun solution and calcined at 850 °C.

To check the distribution of the incorporated metals in the prepared carbon nanofiber matrix, line elemental mapping analysis has been performed; Fig. 5A. As shown in the figure, along with the randomly selected line, nickel, molybdenum and carbon have been detected. Interestingly, the concentration gradients of the two metals are almost similar which concludes that the observed inorganic nanoparticles (Fig. 4) have homogeneous composition. In other words, nickel and molybdenum have a symmetric distribution in the prepared nanofibers which indicates uniform allocation of the active sites in the proposed electrocatalyst. Figure 5B depicts the EDX analysis results, as shown, the results further support the other used analytical techniques and simultaneously confirm the concluded composition of the introduced functional material. As can be seen, EDX analysis reveals presence of nickel, molybdenum and carbon and absence of other elements. Moreover, it could be concluded that nickel is the predominant constituent.

**Figure 6:**
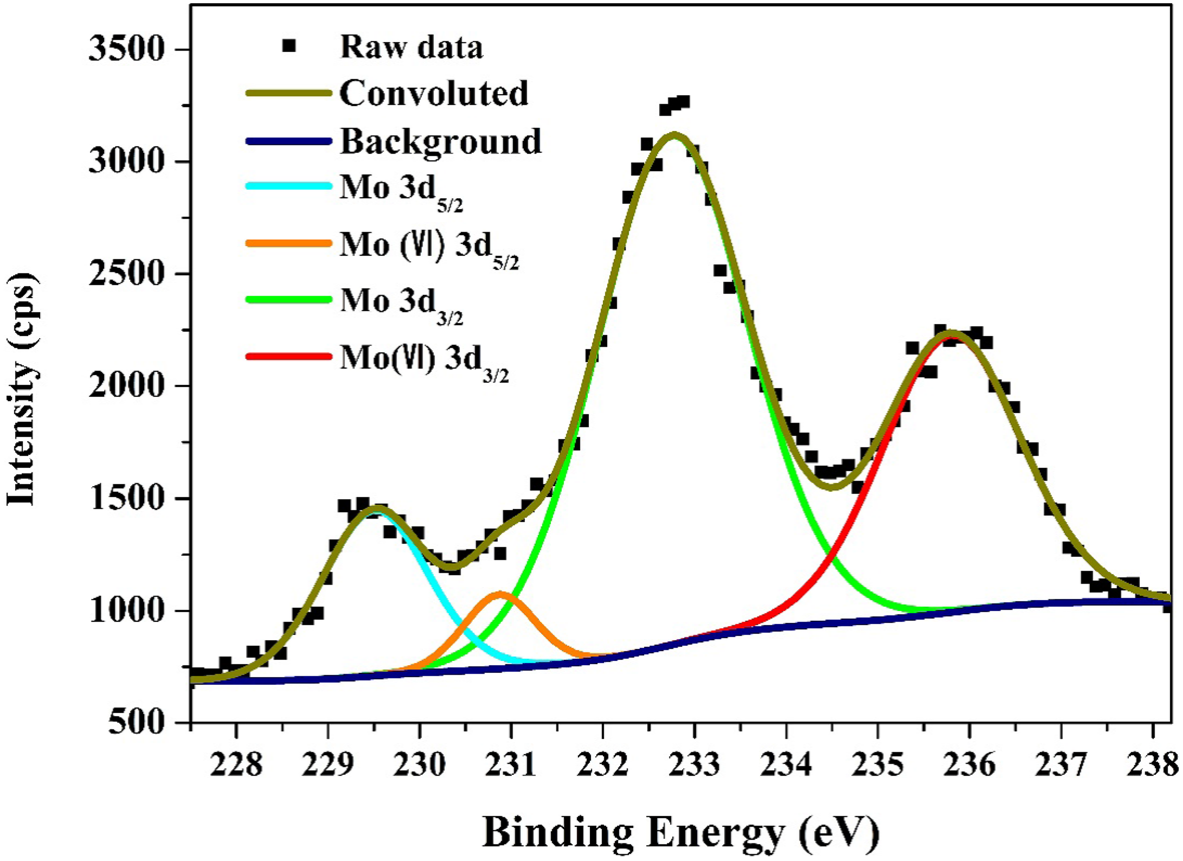
XPS spectra of Mo3d for the prepared Mo_2_C/Ni-incorporated carbon nanofibers obtained from 25 wt% molybdenum chloride electrospun solution and calcined at 850 °C.

Figure 6 demonstrates the XPS spectra of Mo 3d for nanofibers prepared from calcination at 850 °C of electrospun nanofibers obtained from a solution having 25 wt% molybdenum chloride precursor. As shown, the high-resolution Mo 3d spectrum has been deconvoluted into four peaks. The peaks corresponding to Mo 3d_5/2_ (≈ 229.6 eV) and Mo 3d_3/2_ (≈ 232.8), with a spin energy separation of 3.2 eV, demonstrate the characteristic doublets of the Mo^2+^ state of Mo_2_C^47,48^. However, Mo^6+^ representing peaks were also detected (at ≈ 230.9 and 235.8 eV) which concludes that the surfaces of the as-prepared nanofibers are contaminated by MoO_3_ oxide^49^.

From a previous study, it was concluded that carbon content in Ni NPs-incorporated carbon nanofibers prepared by a similar procedure is around 5 wt%^50^. Accordingly, the elemental composition of the produced nanofibers could be summarized in this Table 1.

**Table 1:** Elemental composition of the produced nanofibers.

MoCl_2_ in the initial electrospun solution	Elemental composition of the produced nanofibers (%)		
	Ni	Mo	C
0	95	0	5
5	83.6	10.73	5.67
10	73.77	19.98	6.25
15	65.2	28.05	6.75
25	50.98	41.43	7.59
35	39.67	52.08	8.25

### Electrochemical performance

#### Electroactive service area

Surface activation of the nickel-based is a mandatory to be applicable electrocatalyst for urea oxidation. The activation process is carried out by generation of Ni(OOH) species on the surface through sweeping in a strong alkaline solution or simultaneously with the electrooxidation reactions^51^. The activation process is performed in two main steps which appear as two regions in the voltammograms; the first which is observed at a negative potential region (at ~  − 650 mV) is attributed to the formation of nickel hydroxide^52^:

9 $$ {\text{Ni }} + {\text{ 2OH}}^{ - } \leftrightarrow {\text{Ni}}\left( {{\text{OH}}} \right)_{{2}} + {\text{ 2e}}. $$

It is noteworthy mentioning that the corresponding peak of this reaction is usually very small in the first cycle and vanishes in the subsequent ones^52–54^. At the positive side, the second transformation is done which is associated with appearance of a strong peak due to the oxidation of Ni(OH)_2_ to NiOOH^54^:

10 $$ {\text{Ni}}\left( {{\text{OH}}} \right)_{{2}} + {\text{ OH}}^{ - } \leftrightarrow {\text{NiOOH}} + {\text{ H}}_{{2}} {\text{O }} + {\text{ e}}. $$

Increasing the number of sweeping cycles leads to a progressive increase of the current density values of the cathodic peak due to the entry of OH^−^ into the Ni(OH)_2_ surface layer, which results in a progressive formation of a thicker NiOOH layer^52^. The entrance of OH^-^ into the Ni(OH)_2_ surface layer causes a gradual increase in the current density values of the cathodic peak as the number of sweeping cycles increases, resulting in the creation of a thicker NiOOH layer^52^. Formation of the Ni(OOH) active layer was extensively studied using XPS analysis^51^.

**Figure 7:**
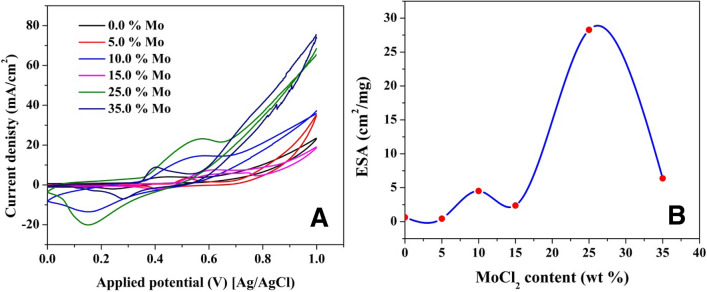
Activation of Mo_2_C/Ni-incorporated carbon nanofibers with different Mo content and prepared at 850 °C (**A**), and influence of the Mo content on the electrochemical surface area (**B**).

The activity of the nickel-based electrocatalysts directly proportions with the amount of the formed active species; the electroactive surface area (ESA). The ESA can be calculated from the cyclic voltammetry of the activation process using the following equation^14,55,56^.

11 $$ ESA = \frac{Q}{mq}, $$

where *Q* (mC) is the charge required to reduce NiOOH to Ni(OH)_2_, *m* (mg) is the nickel amount in the functional catalyst and *q* is the charge associated with the formation of a monolayer from Ni(OH)_2_. Because only one electron is required for the NiOOH → Ni(OH)_2_ transition, *q* can be set to 257 µC/cm^2^^57,58^. *Q* can be determined from the area of the cathodic peak after redrawing the curve as current (mA) versus time (s). The cyclic voltammograms for nanofibers with varied molybdenum contents and calcined at 850 °C are shown in Fig. 7A. The Ni(OH)_2_/NiOOH transition peaks are clearly visible in all formulations, as illustrated.

The effect of molybdenum concentration on the ESA of produced nanofibers is seen in Fig. 7B. Based on the findings, it can be inferred that by optimizing Mo content, the ESA may be significantly improved. The ESA of nanofibers generated from a solution containing 25 wt% molybdenum precursor rose to be 45 times greater than that of Mo-free nanofibers, as indicated; the estimated ESAs for the two formulations were 28.27 and 0.64 cm^2^/mg, respectively. The results also indicated that the slight addition of the proposed co-catalyst does not have a distinguished improvement on the formation of the Ni(OOH) active layer. Numerically, almost similar ESA values were determined from the 0 and 5 wt% samples as shown in the figure. More increase in the MoCl_2_ content in the initial electrospun solution results on a relatively good enhancement in the ESA. Numerically, ESAs generated on the surface of nanofibers obtaining from solutions having 10 and 15 wt% Mo precursor are 4.52 and 2.38 cm^2^/mg, respectively, which are 7 and 3.7 times greater than those created on the surface of Ni/C nanofibers, respectively. However, the relationship between the ESA and the Mo content is not linear as shown. The relationship can be interpreted by a polynomial function as further increase in the co-catalyst precursor more than the optimum content leads to have decreasing in the ESA value. For the 35% sample, the detected ESA value was 6.37 cm^2^/mg. Therefore, it is expected that molybdenum incorporation can enhance the urea electrooxidation process as result of increasing ESA.

#### Urea electrooxidation

**Figure 8:**
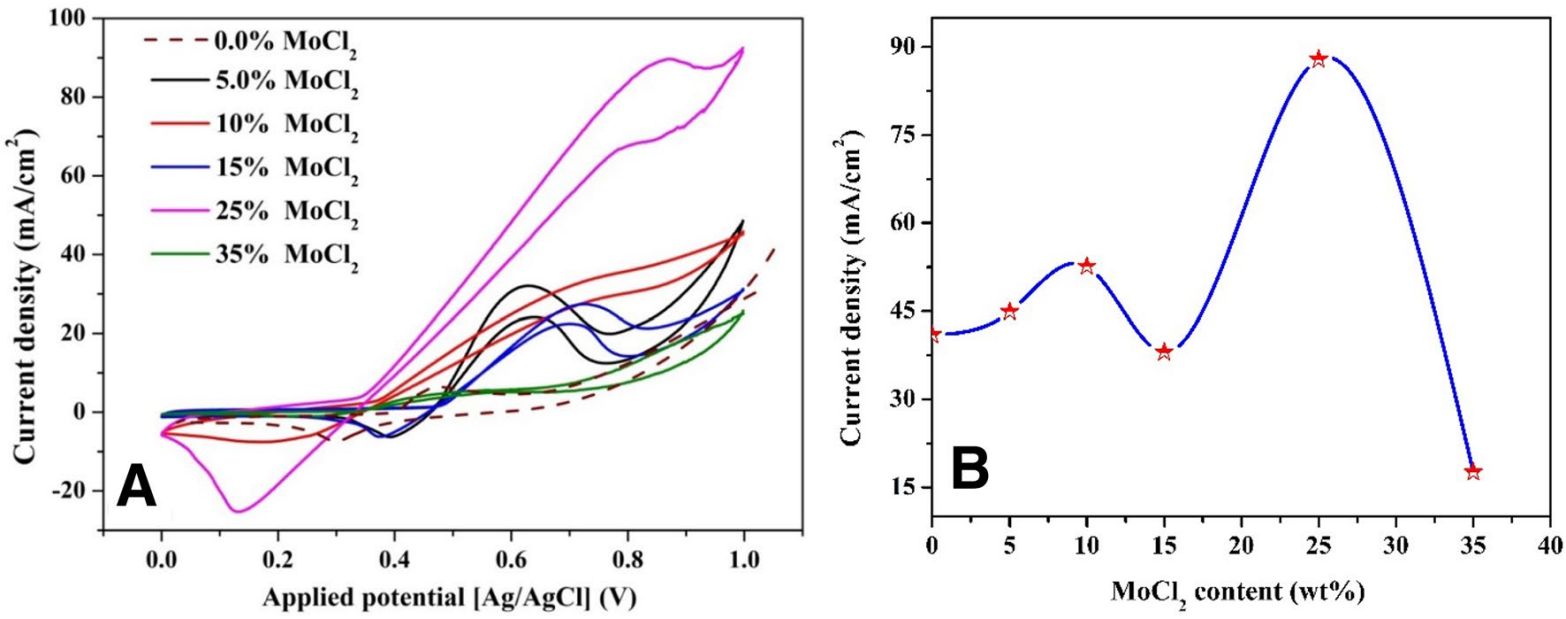
Cyclic voltammograms using the prepared electrodes in presence of 0.33 M urea (in 1.0 M KOH at scan rate of 50 mV/s and 25 °C) (**A**) and influence of molybdenum precursor content in the initial electropsun solution on the maximum current density (**B**).

The performance of the suggested materials in the electrooxidation of urea was tested to scientifically show the impact of the ESA on the electrocatalytic activity of the proposed composites. Figure 8A shows the electroactivity of nanofibers synthesized at 850 °C for urea oxidation (1.0 M urea in 1.0 KOH, scan rate 50 mV/s) at varied Mo concentrations. The nanofibers with the highest ESA (25 wt% Mo), as shown, have the highest activity. Moreover, as shown in Fig. 8B, the observed maximum current density behavior resembles that of the ESA (Fig. 7B). This finding indicates that electrooxidation of urea over the proposed electrodes directly proportions with the ESA value mimicking the alcohols oxidation process.

In contrast to alcohols, urea oxidation reactions are complicated so no a confident mechanism could be assured. Many researchers tried to explain the urea oxidation mechanism. Among these trials, the density functional theory (DFT) reported by Botte et al. is the most reliable^59^. In that report, the authors have suggested three possible paths for urea dissociation. The most important finding, they concluded that carbon dioxide desorption is the common rate-controlling step. Carbon dioxide adsorption negatively affects the redox reaction reversibility. Therefore, it can be claimed that the generated current density is not the unique parameter governing the performance of the electrocatalyst. Beside the generated current density, the difference between the redox peaks potentials (Δ*E*_*p*_ = ǀ*E*_*pa*_ − *E*_*pc*_ǀ) is another important characteristic for the effective electrocatalyst because it is related to the electrode reversibility. In the completely reversible redox reactions, the redox peaks potentials difference is independent on the sweep speed and possesses a very small value verifying this equation^60^.

12 $$\Delta {E}_{p}=\frac{0.0565}{n},$$

where *n* is the number of electrons sharing in the reactions; equals 6 for urea oxidation reaction. However, satisfying this equation is an ideal hope and cannot be achieved in the practical situation; complete reversible process is an ideal case. Another important finding which can be extracted from the obtained results in Fig. 8A is the location of the redox peaks potentials for the samples revealing the highest current densities. As shown, the cathodic peaks corresponding to the nanofibers prepared from initial solutions having 10 and 25 wt% molybdenum chloride are close to the Ni(OH)_2_/Ni(OOH) one (Fig. 7A). This finding is interesting as it indicates a self-regeneration of the used electrodes^52^. Moreover, 5 wt% shows the minimum difference between the cathodic and anodic peak potentials which reflects maximum reversibility among all other formulations except the Mo-free electrode.

Our previous detailed study concluded that activation of the active Ni-based electrocatalysts can be performed simultaneously with the oxidation reaction^51^. Considering that the Ni(OOH) is a reactant, this characteristic is important as it suggests sustainable electrode. For other formulations, the cathodic peaks are close to urea oxidation assigned in many reports^7,61^. Although urea oxidation is mainly anodic reaction, the cathodic peaks, which have usually lower current densities compared to the anodic peaks, represent oxidation of some intermediates^59^. However, for the 10 and 25 wt% samples, the cathodic peaks are broad and can be attributed to both of Ni(OH)_2_/Ni(OOH) transformation and urea oxidation. To properly evaluate the introduced electrocatalyst, the performance was compared with some recently reported functional materials in Table 2.

**Table 2:** Performance comparison between reported electrocatalysts and the introduced Ni–Mo_2_C–carbon nanofibers toward urea oxidation.

No	Electrocatalyst	Current density mA/cm^2^	References
1	Hierarchical Ni(OH)_2_–NiS nanosheets on carbon cloth (Ni(OH)_2_–NiS–CC)	87.5	^62^
2	Co–Ni(OH)_2_ nanosheet array on Ni foam electrodes	59.7	^63^
3	NiS-decorated carbon nanofibers	37.5	^64^
4	Ni_2_P nanoflower-supported nickel foam	750	^65^
5	Ultrathin NiO nanosheets-decorated ultrafine Rh nanocrystals	22.5	^66^
6	Tri-metallic MnNiFe alloy nanoparticles (Mn_0.5_Ni_2.0_Fe_0.5_/rGO)	2.86	^67^
7	Nickel-molybdenum oxide nanorods Ni/Mo	19	^31^
8	MoS_2_, NiS, and Co_3_S_4_ MCNS/reduced graphene oxide composite	43	^68^
9	Ni-decorated multi-layers graphene sheets	81.75	^69^
10	LNF-C/MWCNT	1.246	^70^
11	Ni–Cu/ZnO@MWCNT	30.02	^71^
12	NiSn-incorporated carbon nanofibers	77 (at 30 °C) 175 (at 55 °C)	^10^
13	Pd–Ni/C	80	^72^
14	Ni–Cd carbon nanofibers	67.2	^29^
15	Nickel cobalt phosphate (NiCoPO)	70	^73^
16	Tungsten carbide in molten salt (Ni–W_x_C/C )	50.31	^74^
17	Mesoporous nickel phosphide (Ni–P)	70	^24^
18	Nickel phosphates nanotube	32	^75^
19	CoS_2_ nanoneedle array grown on Ti mesh (CoS_2_ NA/Ti)	10	^76^
20	NiSn/CNFs	5	^10^
21	Nickel phosphide (NiP)	22.26	^77^
22	Ni/carbon black	8	^78^
23	Ni-decorated N-doped three-dimensional graphene	12.5	^78^
24	Ni(OH)_2_/polypyrrole/graphene nanosheets	1.25	^79^
25	Ni_1.5_Mn_1.5_O_4_	6.90	^80^
26	Sodium nickel fluoride	8.50	^81^
27	Tungsten carbide-incorporated carbon nanofibers	37.75	^22^
28	NiS@CNFs	40.46	^64^
29	Ni/Mo_2_C-incoporated carbon nanofibers	44.9 (Mo 10.7%)	This study
		52.6 (Mo 19.9%)	
		30.1 (Mo 28.1%)	
		87.9 (Mo 41.4%)	
		17.6 (Mo 52.1%)	

#### Effect of concentration

**Figure 9:**
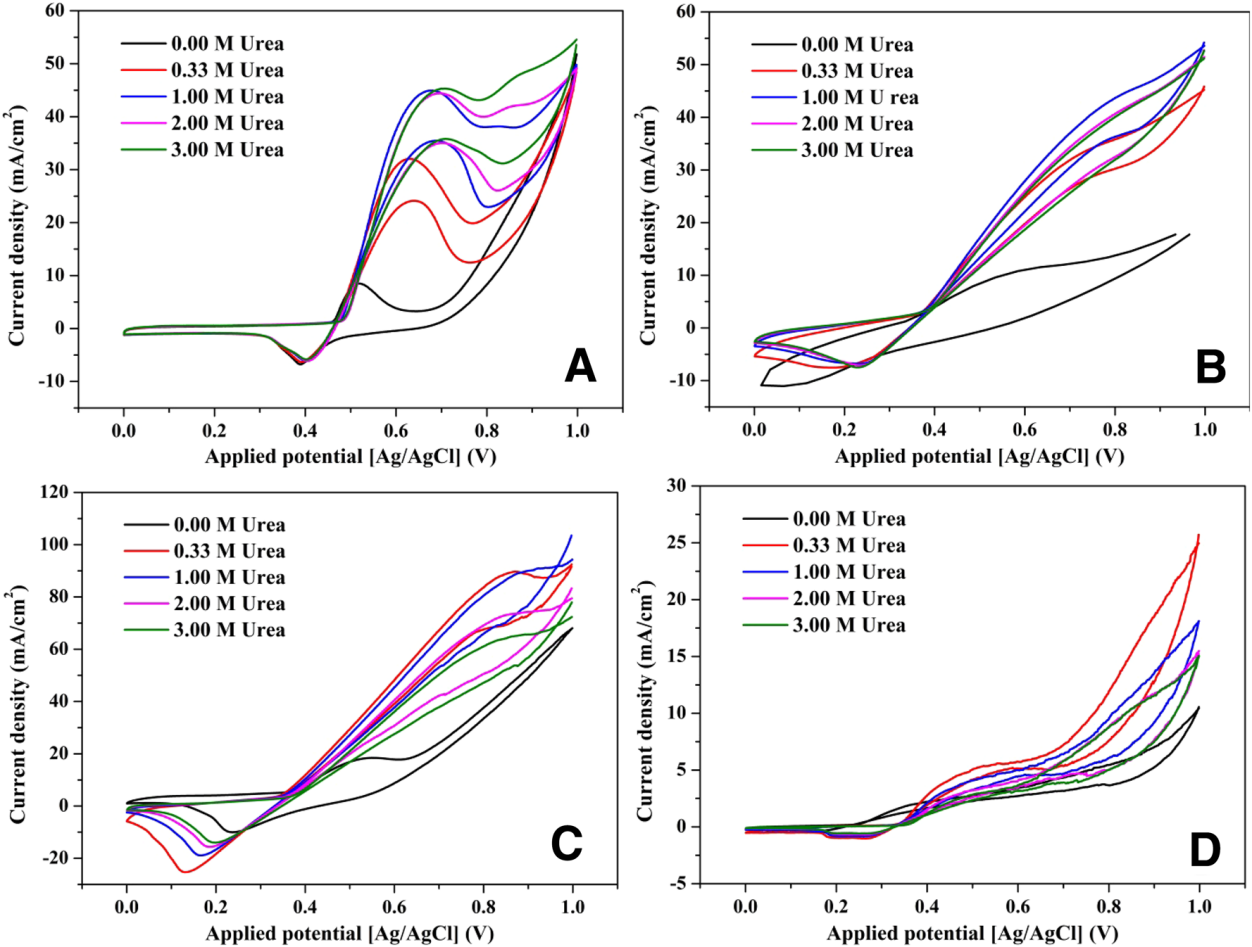
Electrocatalytic activity of the proposed NiMo-incorporated carbon nanofibers toward urea oxidation at different Mo precursor contents; 5 (**A**), 10 (**B**), 25 (**C**); and 35 (**D**), at 850 °C and 50 mV/s scan rate and 25 °C.

Like any non-zero order chemical reaction, the concentration of the main reactant (urea) strongly affects the reaction rate. However, this impact depends on the catalytic activity of the used catalyst. Figure 9 displays the cyclic voltammograms for different prepared catalysts at various urea concentrations. For most of the investigated formulations, it clearly appears that addition of urea resulted in a sharp increase in the current density which confirms the good electrocatalytic activity of these electrodes toward urea oxidation process. Mass transfer operation distinctly affects the kinetics of the heterogeneous catalytic reactions. In this regard, increasing the reactants concentration does not only enhance the reaction rate but also improve the mass transfer process. However, the rate-limiting step for the whole process can be mass transfer of the reactant(s) or the reaction(s) rate(s) or the mass transfer rate of the product(s). Therefore, increasing the reactant(s) concentration can show an influence when it represents the rate-limiting step for the whole process, after that a negligible or even negative impact might be observed^23,26^. These hypotheses are proved in Fig. 9. In details, as shown in Fig. 9A, for the nanofibers obtained from sol–gel having 5 wt% MoCl_2_, increasing urea concentration from 0.33 to 1.0 M led to have a high jump in the anodic peak current density. More increase in urea concentration did not show a noticeable change in the peaks current density, but, a considerable increase in the current can be observed at higher applied potential with increasing the concentration. Therefore, for these nanofibers, it can be claimed that urea concentration is the rate-limiting process at high range of concentration which reflects high electrocatalytic activity of this electrode. There is a slight change in the situation in case of 10 wt% electrode as shown in Fig. 9B. As shown, there is a realizable increase in the current density upon increase the concentration of urea from 0.33 to 1.0 M. However, 1.0 M concentration stilled the predominant within the used potential window; 2.0 and 3.0 M urea concentrations demonstrated equal and lower (compared to 1.0 M) current densities.

Although, the electrode prepared from the solution having 25 wt% molybdenum precursor revealed the maximum current density, the increase of urea concentration from 0.33 to 1.0 M did not result in an observable increase in the current density as shown in Fig. 9C. Moreover, more increase in the reactant concentration led to have a negative influence in the detected current density. As a high current density was obtained, it is believed that the rate-limiting step in case of utilizing this electrode is the mass transfer of the products. In other words, low desorption rate of CO_2_ gave rise to decrease the reaction rate which was translated into getting relatively small current density at high urea concentrations. For the last sample (35 wt% MoCl_2_; Fig. 9D), the results confirm the low activity of the proposed nanofibers at this composition. It is noteworthy mentioning that, previous reports indicated that the generated current density directly proportions with the urea content at low concentration range (< 1.0 M urea)^82,83^. However, it is important to say that there is no conflict between the results in this study and the previous reports. The optimum concentration observed in this study represents the maximum concentration validates the linear relation between the urea concentration and the generated current density.

#### Effect of synthesis temperature

**Figure 10:**
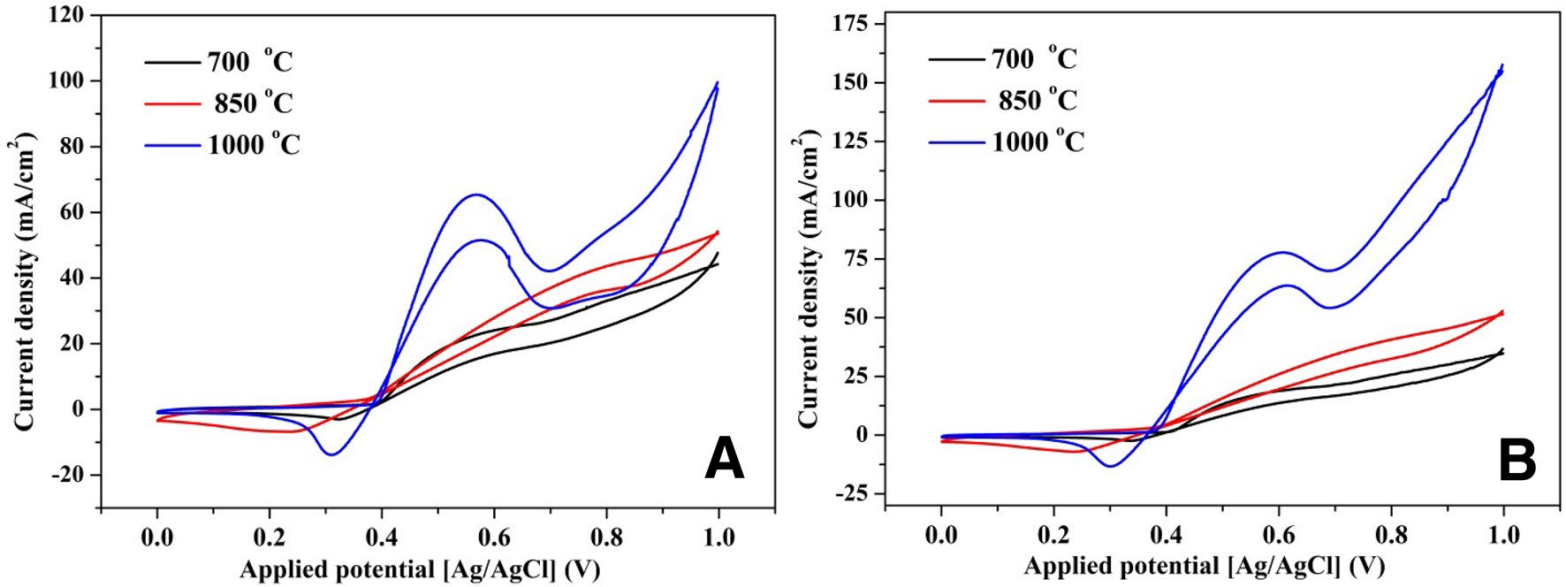
Influence of the calcination temperature on the electrocatalytic activity of the proposed NiMo-incorporated carbon nanofibers (10% Mo precursor) toward urea oxidation at different urea solution concentration, 1 M (**A**) and 2 M (**B**) at 50 mV/s scan rate and 25 °C.

It is known that the catalytic activity of the solid materials depends mainly on the surface electronic structure which is usually a follower to the material crystallinity. Consequently, effect of the synthesis temperature of the catalyst on the electroactivity has been investigated in Fig. 8. The results display the obtained voltammograms in case of utilizing 10 wt% electrode prepared at different temperatures using two concentrations of urea solutions; 1.0 M (Fig. 10A) and 2.0 M (Fig. 10B). As it can be plainly concluded, preparing the proposed nanofibers at high temperature (1000 °C) mightily enhances the electroacatalytic activity of the proposed functional material in two terms; obtaining high current density and clear appearance of the urea oxidation peak. This finding can be attributed to the high crystallinity of the inorganic material prepared at this elevated temperature. On the other hand, increasing the treatment temperature from 700 to 850 °C did not reveal a high difference in the electrocatalytic activity.

#### Electrode stability

**Figure 11:**
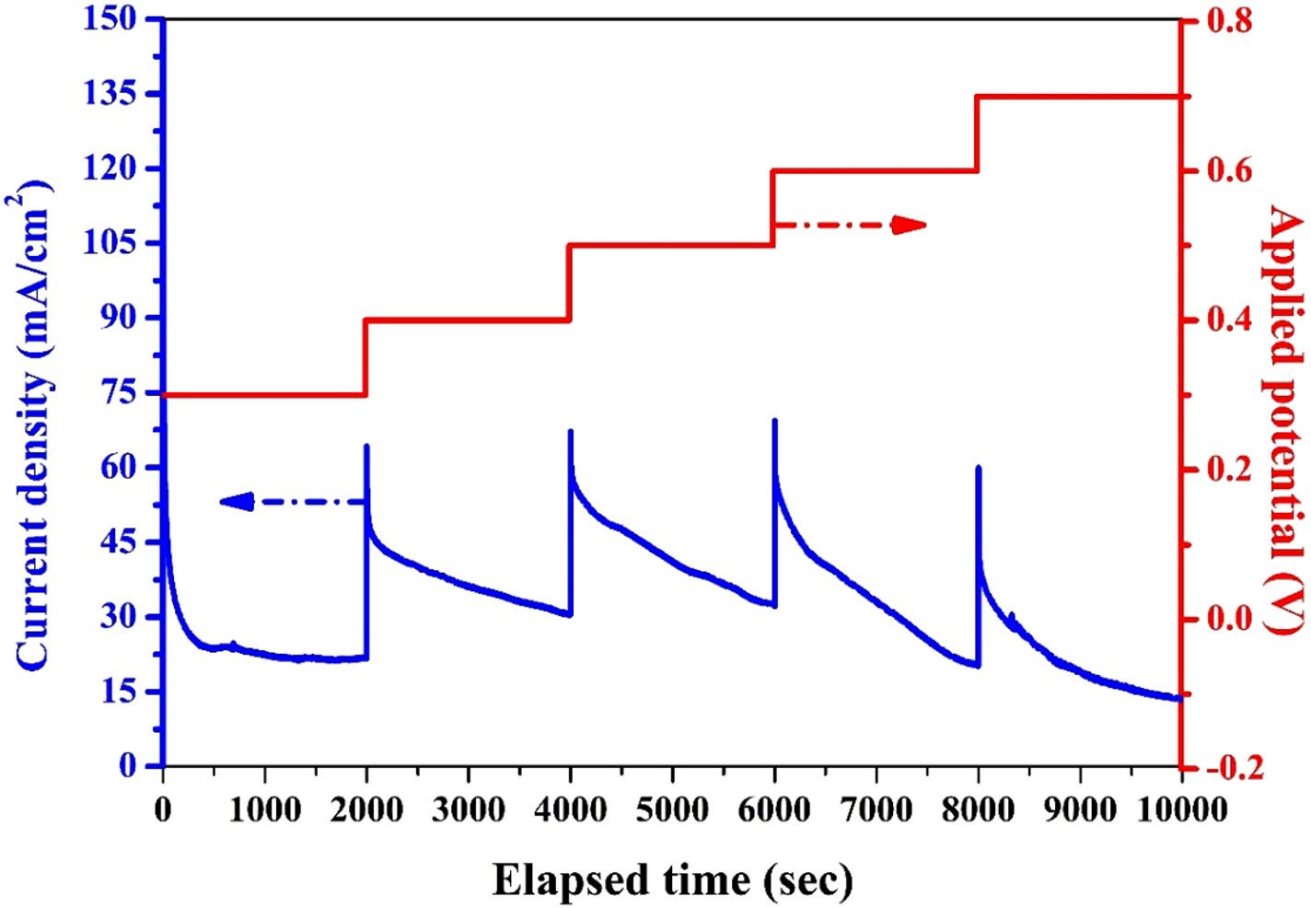
Chronoamperometery analysis at a potential of 0.6 V for the nanofibers obtained from sol–gel having 25 wt% MoCl_2_ and thermally treated at 1000 °C in presence of 1.0 M urea (in 1.0 M KOH) at room temperature.

Stability is an important parameter for the practical electrodes. Figure 11 depicts the chronoamperometry analysis for the electrode revealed the highest performance; 25 wt% prepared at 1000 °C. The applied potential in this analysis was increased in a stepwise way starting from 0.3 V until 0.7 V with a holding time of 2000s at every step; the total elapsed time was 10,000 s. The results confidently support the stability of the used electrode. Briefly, as the analysis was conducted in a stagnant solution, the sharp decrease in the current density at the start of every step is attributed to the exhaustion of the urea in the zone nearby the electrode active surface area, and the inability of the mass transfer process to compensate the decrease in the reactant concentration. However, increasing the potential window led to increase the generated current density which can be translated as good stability of the prepared electrode.

#### Reaction kinetics

**Figure 12:**
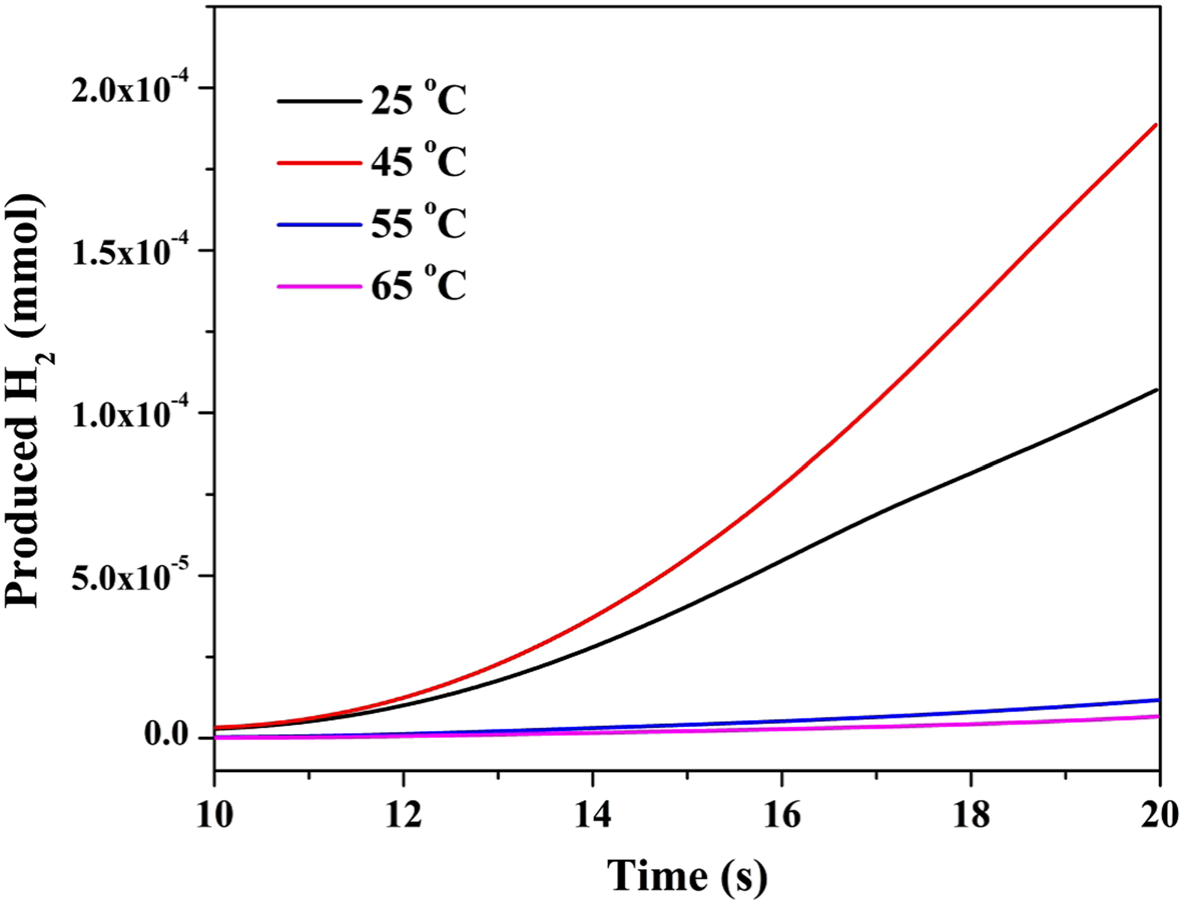
Influence of the reaction temperature on the hydrogen production rate using the proposed composite (15% Mo precursor) calcined at 850 °C at 0.05 V/s scan rate.

In the homogeneous chemical reactions, Arrhenius equation is applicable because rising the temperature leads to increase the kinetic energies of the reactants molecules which results in increasing the collisions between the reactants. Consequently, the reaction rate improves with increasing the medium temperature. However, in the case of the heterogeneous catalytic reactions, enhancement the reactants molecules acceleration can give negative effect as it can lead to running the reactants away from the catalyst surface^84^; analogy to the adsorption process. Figure 12 explains the effect of reaction temperature on the dissociation of urea. As shown, increase the temperature from 25 to 45 °C reveals positive influence as the dissociation rate increases. However, more increase in temperature to 55 °C led to a sharp decrease in the reaction rate. Moreover, at 65 °C, urea oxidation process was almost annihilated. Therefore, it can be claimed that urea oxidation reaction using the proposed electrocatalyst does not follow Arrhenius equation. Kinetic calculations have been done to estimate the reaction constant at each temperature. Table 3 summarizes the obtained data. As shown, at 45 °C (318 K), the reaction constant is close to unity while it diminishes to be very small (0.019/s) at the highest applied temperature; 65 °C.

**Table 3:** Urea electrooxidation reaction constants at different reaction temperatures.

Temperature (K)	298	318	328	338
Reaction constant (s^−1^)	0.457	0.985	0.046	0.019

#### Influence of the scan rate

**Figure 13:**
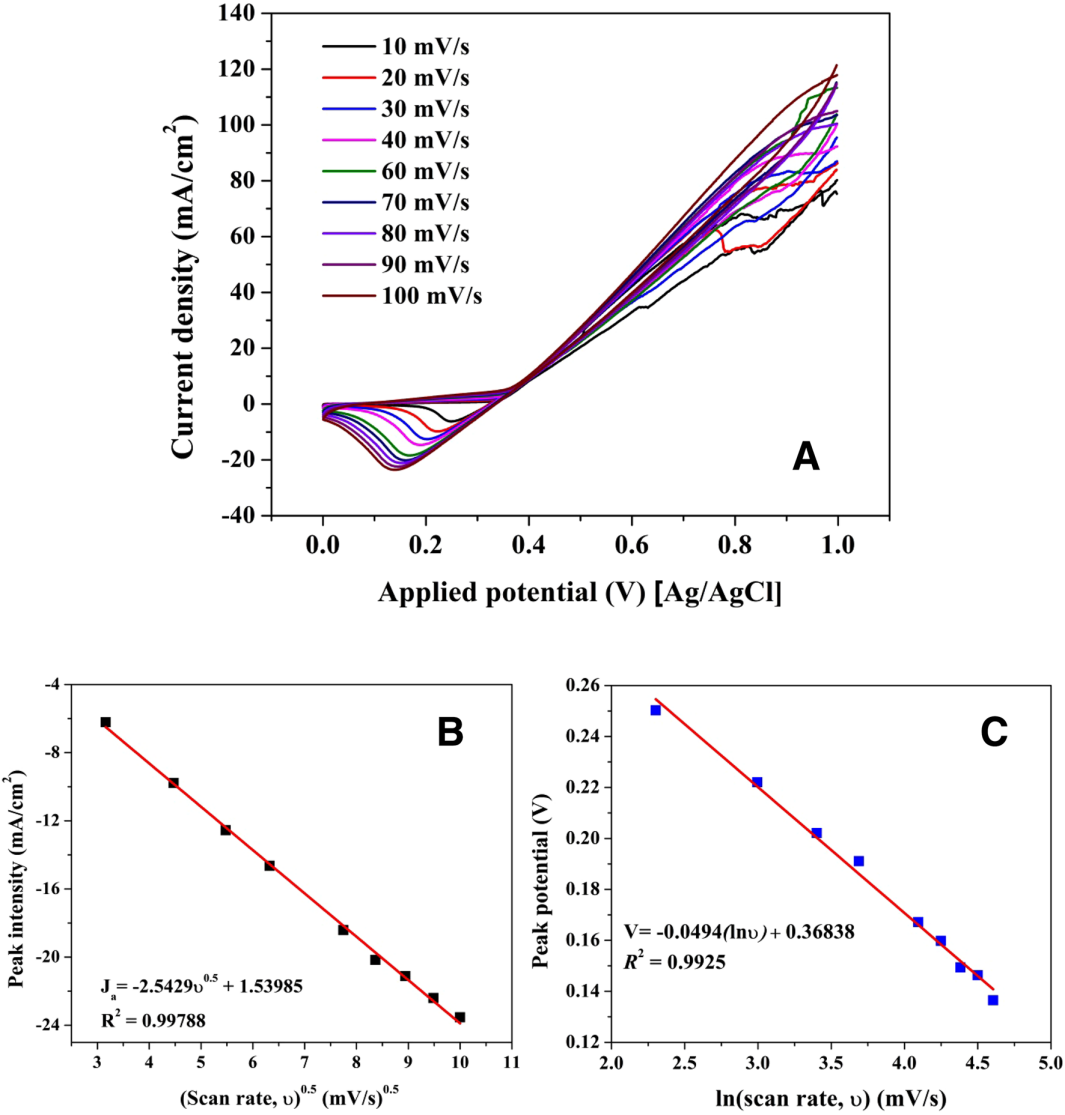
(**A**) Cyclic voltammograms of Mo_2_C/Ni-incorporated carbon nanofibers (25 wt%) at different scanning rates in 0.33 M urea (in 1.0 M KOH); (**B**) plots of J_pa_ vs. scanning rates from (**A**); (**C**) Laviron’s plots of cathodic peak potential vs. ln*v* from (**A**).

Figure 13A presents the influence of scanning rates evaluated using CV in presence of 0.33 M urea (in 1.0 M KOH) using the nanofibers obtained from 25 wt% MoCl_2_ solution. As it was previously explained, the cathodic peak represents the Ni(OH)_2_/Ni(OOH) transformation^85^. The reduction peak’s current density (*j*_ca_) decreased continuously with the scanning rates. *j*_*ca*_ value presented an excellent linear relationship against ν^0.5^ in the range from 10 to 100 mV/s (*R*^2^ = 0.9978) in Fig. 13B, indicating that the electrochemical characteristic of the diffusion process of OH^-^ from the solution to the used electrode was a typical diffusion-controlled process^86^. In addition, by the increasing of scanning rates, the peak potential (*E*_*pc*_) moved in the direction of the negative potential. Figure 13C shows that *E*_*pc*_ depended linearly on ln(*ν*) (10 to 100 mV/s) with the equation of *E*_*pc*_ (V) = 0.36838 − 0.0494 ln (*ν*) (V/s) (*R*^2^ = 0.9925) which shows very good matching to Laviron theory for thin-layer quasi-reversible electrochemical process^87^.

## Conclusion

Calcination of electrospun mates composed of poly(vinyl alcohol), nickel acetate and molybdenum chloride under vacuum atmosphere leads to decomposition of the metallic ingredients to zero-valent nickel and molybdenum carbide nanoparticles incorporated in amorphous graphite nanofibers. The proposed composite nanofibers can be exploited as efficient and stable electrocatalyst for urea oxidation process when the Mo content is optimized. To get maximum urea dissociation rate with in-vivo electrode regeneration, the molybdenum precursor in the initial electropun solution has to be kept at 25 wt% with respect to the nickel acetate. Synthesis temperature is a critical factor as preparing the proposed electrocatalyst composite at 1000 °C strongly enhances the catalytic activity toward urea. Unlike the conventional chemical reactions, increasing the reaction media temperature does not generally enhance the reaction rate; the highest urea dissociation rate can be achieved if the temperature is maintained at 45 °C. Finally, it is highly recommended to use the suggested Mo_2_C/Ni‒incorporated carbon composite in nanofibrous morphology to get high performance.

## Data Availability

The datasets used and/or analyzed during the current study available from the corresponding author on reasonable request.
